# Editorial: Pathways and Processes Underpinning Axonal Biology and Pathobiology

**DOI:** 10.3389/fnmol.2022.883244

**Published:** 2022-03-23

**Authors:** Pabitra K. Sahoo, Dianna E. Willis, James N. Sleigh

**Affiliations:** ^1^Department of Biological Sciences, University of South Carolina, Columbia, SC, United States; ^2^Burke Neurological Institute, White Plains, NY, United States; ^3^Feil Family Brain & Mind Research Institute, Weill Cornell Medicine, New York, NY, United States; ^4^Department of Neuromuscular Diseases and UCL Queen Square Motor Neuron Disease Centre, UCL Queen Square Institute of Neurology, University College London, London, United Kingdom; ^5^UK Dementia Research Institute, University College London, London, United Kingdom

**Keywords:** axonal translation, axonal transport, nerve injury, nerve regeneration, neurotrophins, Wallerian degeneration

Neurons are highly polarized cells with axons that can extend beyond a meter. Axons provide long-range connections between somas and target cells, permitting rapid electrical communication. Given these distances, axons require a continuous supply of organelles, mRNAs, and proteins. This is achieved through bi-directional cargo trafficking along microtubules by motor proteins in the process of axonal transport; however, *in vivo* axonal transport rates are well below 10 μm/s (Tosolini et al., 2021), thus additional mechanisms are required for distal axons to efficiently respond to environmental stimuli. Consequently, local translation of readily-available transcripts provides an important mechanism to overcome distance constraints and allow proteome modification (Dalla Costa et al., 2021). Nevertheless, these vital processes are not independent since mRNA must travel from the nucleus to translation sites. In fact, transport and translation are intricately linked through a process called “hitchhiking,” whereby mRNAs are co-transported with organelles through tethering of RNA-binding proteins (RBPs) for on-demand axonal translation (Vargas et al., 2022). Accordingly, disruption of transport or translation in axons can impair neurodevelopment and drive neurodegeneration (Costa and Willis, 2018; Sleigh et al., 2019). Nonetheless, despite recent developments in axon-specific methods (Farias et al., 2019; Surana et al., 2020), it is often unclear whether perturbations are a cause or consequence of neurodegeneration.

With this special issue, we aim to showcase recent advances in axon biology to stimulate a field poised to generate a comprehensive understanding of pathways and processes critical to axonal integrity. We present ten articles covering themes of transport/translation, degeneration, and nerve injury/regeneration (Figure 1).

**Figure 1:**
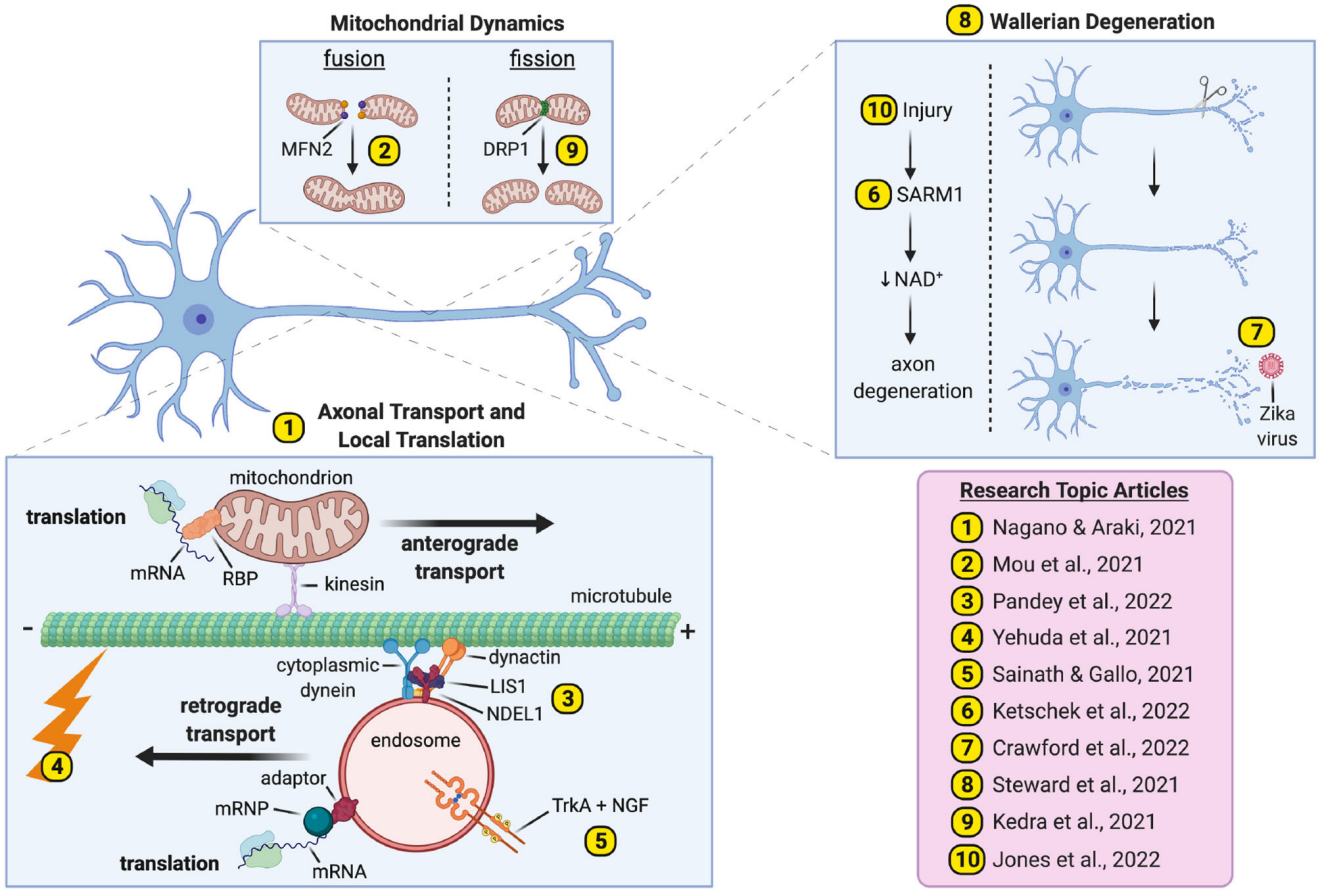
Pathways and processes underpinning axonal biology and pathobiology. We present ten papers covering major themes of axonal transport and translation (Articles 1–5), Wallerian degeneration (Articles 6–8) and nerve injury/regeneration (Articles 9–10). *mRNP*, mRNA-containing ribonucleoprotein; *RBP*, RNA-binding protein. The figure was created with BioRender (https://Biorender.com).

Nagano and Araki present a review discussing the significance of axonal transport and translation to pathogenesis of neurodegenerative diseases. After introducing the physiological importance of these processes, evidence linking their impairment to neurodegeneration is reviewed. It is highlighted that many dysregulated axonal transcripts encode cytoskeleton components of growth cones/synapses, indicating that these mRNAs are required for formation and function of axon terminals.

Mou et al. present a human motor neuron model for mitofusin-2 (MFN2) deficiency. MFN2 mediates mitochondrial fusion and dominantly inherited *MFN2* mutations cause Charcot-Marie-Tooth; however, the mechanisms driving peripheral neuropathy remain unresolved. To address this, *MFN2* was knocked-down and shown to impair morphology, function, and trafficking of axonal mitochondria. Additionally, MFN2-deficient motor neurons displayed increased phosphorylated neurofilament inclusions, as well as diminished motor protein availability.

*LIS1* and *NDEL1* stimulate dynein activity; however, their role in mitochondrial transport remains unclear. Pandey et al. thus evaluated mitochondria in sciatic axons of *LIS*1^+/−^ mice and showed them to be more prevalent than in controls, replicating a disruption in rats caused by dynein inhibition. *LIS1* and *NDEL1* knockdown in rat sensory neurons perturbed physiological morphology, percentage motility and transport speeds of axonal mitochondria. Conversely, *LIS1* overexpression enhanced mitochondrial trafficking.

The axonal cytoskeleton provides the extensive microtubule network along which motor proteins transport cargoes; however, the effects of neuronal activity on this structure remain understudied. Yehuda et al. thus electrically stimulated mouse sciatic nerves prior to nerve excision and electron microscopy. This short, physiological excitation caused clear reductions in microtubule and neurofilament density, indicating that neuronal activity can rapidly depolarize cytoskeletal structures, likely to facilitate morphological changes like axon extension/growth.

Neurotrophins bind Trk receptors on neurons (e.g., NGF preferentially signals through TrkA) to activate homeostatic pathways, including PI3K-Akt. To better understand spatiotemporal aspects of this process, Sainath and Gallo studied the NGF-TrkA axis and PI3K-Akt signaling in mouse sensory neurons. NGF stimulated PI3K-Akt activity preferentially at sites populated with axonal mitochondria. Moreover, this initial activation was oxidative phosphorylation-dependent, but not glycolysis-dependent, differing from longer-term observations, suggesting temporal distinctions in bioenergetic requirements of neurotrophin signaling.

Caused by injury, Wallerian degeneration (WD) is a pathway of programmed axon death driven by SARM1-mediated depletion of axonal NAD^+^. Ketschek et al. studied *Sarm1* knockout mice to assess its role in axon branching. Sarm1 disruption increased axonal branching in skin and cultured sensory neurons. Moreover, through live imaging, *Sarm*1^−/−^ axons were shown to display altered cytoskeletal dynamics, decreased axonal mitochondrial motility, and increased actin-patch formation leading to enhanced filopodia establishment.

Crawford et al. generated mouse myelinating-spinal cord cultures to study SARM1 function in Zika virus (ZV) infection. ZV causes axon degeneration *in vitro* and is associated with congenital neurological complications, although the etiology is unclear. To evaluate WD involvement, neurons were cultured from *Sarm1* knockout mice before ZV infection. ZV caused a rapid, but Sarm1-independent, reduction of NAD^+^; however, *Sarm1* loss prolonged survival of neuronal processes, suggesting a role in ZV-driven pathogenesis.

WD is dramatically delayed in *Wld*^*s*^ mice by up to 2 weeks but is less well studied in the CNS than PNS. To combat this, Steward et al. combined WD-eliciting lesions of the entorhinal cortex with electron microscopy to assess synaptic degeneration in the dentate gyrus. Expectedly, synapse loss in *Wld*^*s*^ mutants was substantially delayed. Less predictably, however, *Wld*^*s*^ mice displayed dendritic spine hypertrophy and enlarged post-synaptic membrane specializations, similar in effect to long-term potentiation.

Mitochondria aid WD execution, while their dysfunction can also trigger WD. Their putative role(s) in nerve injury response is less well understood. Using a mouse spinal cord injury model, Kedra et al. thus analyzed mitochondrial response in corticospinal tract axons. Injury caused increased mitophagy and persistent mitochondrial fragmentation, which was dependent on both calcium uptake and Drp1, a GTPase required for mitochondrial fission. Nerve injury therefore caused increased mitochondrial fission in CNS neurons.

Diabetes can cause peripheral neuropathy through undefined mechanisms, although impaired nerve regeneration may contribute. Jones et al. used rats to assess the ability of diabetic peripheral nerves to regenerate post-injury. Impaired nerve regeneration in diabetic rats was linked to reduced axonal mRNA transport caused by reduced availability of the RBP ZBP1. ZBP1 overexpression rescued regrowth in diabetic neurons post-injury, indicating that perturbed ZPB1-mediated transcript mobilization into regenerating axons contributes to diabetes-induced neuropathy.

In summary, the ten articles presented in this collection cover a breadth of topics converging on mechanisms governing axonal homeostasis. We believe this Research Topic will be of interest to researchers working on areas including developmental neurobiology, neuronal signaling and neurological diseases.

## Author Contributions

JS drafted the manuscript and figure. All authors contributed to the completion of the work and approved its submission.

## Funding

This work was supported by South Carolina Spinal Cord Injury Research Fund (PS), the Burke Foundation (DW), and the Medical Research Council Career Development Award (MR/S006990/1) (JS).

## Conflict of Interest

The authors declare that the research was conducted in the absence of any commercial or financial relationships that could be construed as a potential conflict of interest.

## Publisher's Note

All claims expressed in this article are solely those of the authors and do not necessarily represent those of their affiliated organizations, or those of the publisher, the editors and the reviewers. Any product that may be evaluated in this article, or claim that may be made by its manufacturer, is not guaranteed or endorsed by the publisher.
